# My story of sex, gender, and women's health in a pandemic*

**DOI:** 10.1111/imr.13111

**Published:** 2022-06-20

**Authors:** Sabra L. Klein

**Affiliations:** ^1^ W. Harry Feinstone Department of Molecular Microbiology and Immunology The Johns Hopkins Bloomberg School of Public Health Baltimore Maryland USA; ^2^ Department of Biochemistry and Molecular Biology The Johns Hopkins Bloomberg School of Public Health Baltimore Maryland USA; ^3^ Department of International Health The Johns Hopkins Bloomberg School of Public Health Baltimore Maryland USA; ^4^ Department of Medicine Johns Hopkins School of Medicine Baltimore Maryland USA

**Keywords:** COVID‐19, inflammation, influenza, pregnancy, sex difference

## Abstract

After more than 20 years of studying sex differences in viral pathogenesis and immunity to vaccines, the COVID‐19 pandemic provided me with a unique opportunity to raise awareness about biological sex differences. The scientific community and public, alike, embraced the clinical and epidemiological data and supported inquiries into how males are twice as likely to be hospitalized and die from COVID‐19. Immunological changes associated with pregnancy also contribute to worse outcomes from COVID‐19. Collectively, we are finding that inflammation is a critical mediator of worse outcomes for males and pregnant females. The pandemic gave me a platform to discuss and address sex differences on a bigger stage, but two decades of studies working with other viruses prepared me for this moment in history.

Early data emerging out of Wuhan, China from late 2019 and early 2020 showed that men were more likely to be hospitalized and die from COVID‐19 than women.^1^ The trend continued as SARS‐CoV‐2 spread throughout Europe.^2^ As the virus took hold in the United States, reports from New York, one of the first states to disaggregate data separately for males and females, mirrored what was seen elsewhere in the world—men were at least twice as likely as women to be admitted into the intensive care unit (ICU) and die from COVID‐19.^3^ We still do not know the cause, but this likely reflects an interaction between biological sex and gender,^4^, ^5^ which further intersects with age,^6^ comorbidities,^2^ and social determinants of health.^7^, ^8^

I had been studying sex differences in susceptibility to infectious diseases for more than 20 years at the start of the pandemic.^9^ In fact, that was the topic of my postdoctoral fellowship. Even in the early years of my career, I had studied sex differences in infectious diseases in human populations,^10^ reservoir hosts,^11^ and mathematical S‐I‐R models.^12^ I had worked with RNA viruses characterizing the mechanisms of sex differences in acute disease,^13^, ^14^, ^15^ virus persistence,^16^, ^17^, ^18^ transmission,^19^, ^20^ and protection following vaccination.^21^, ^22^, ^23^ I had studied how sex steroids,^24^, ^25^, ^26^ aging,^27^, ^28^ pregnancy,^29^, ^30^ and behavior^19^, ^20^ affect the dynamics of viral pathogenesis differently for males compared with females. When the COVID‐19 pandemic was declared, I was armed with decades of knowledge to help me interpret the real‐time epidemiological and clinical data being reported from around the world. In retrospect, I was similarly prepared during the 2009 H1N1 pandemic.^31^, ^32^ While the world was still debating whether COVID‐19 was a pandemic and whether we should be wearing masks, I wrote and submitted newspaper op‐eds throughout the early spring of 2020. All were rejected as I was told that my focus on sex differences was premature. I contacted journal editors in chief, many of whom I knew because these were journals where I was an editor. They approved and fast‐tracked commentaries from me, my collaborators, and trainees.^33^, ^34^, ^35^ Real‐time surveillance kept illustrating that across the adult life course, males were being hospitalized at greater rates than females. Finally, I was invited to write a commentary about our hypothesized mechanisms for how males and females were responding differently to SARS‐CoV‐2.^4^ We illustrated that sex differences in immunity can be mediated by direct effects of sex chromosome complement and X‐linked genes that exhibit dosage effects, differential expression of autosomal genes, sex steroids signaling in immune cells to transcriptionally regulate immune responses, or a combination of these sex‐related factors.^4^ We highlighted that the SARS‐CoV‐2 virus could enter cells from males more readily than cells from females because *ACE2* is an X chromosome‐encoded gene that is downregulated by estrogens.^36^ Previous work from our laboratory and others have shown that innate sensing of viral RNA by the TLR7 pattern recognition receptor is sex‐biased. The *TLR7* gene, which is X‐linked and escapes X‐inactivation resulting in greater expression in female immune cells, has been linked to sex differences in autoimmunity^37^, ^38^ and vaccine outcomes.^21^ There also is greater production of IFNα from plasmacytoid dendritic cells from adult females than males,^39^, ^40^ an effect modulated by sex steroids,^41^, ^42^, ^43^ which could contribute to faster innate immune cell recognition of SARS‐CoV‐2 and downstream signaling in cells from females than males. Elite laboratories began taking notice and either reporting^44^ or mechanistically studying^45^ sex differences. Major newspapers, media outlets, and podcast hosts began interviewing me and wanting to know more about sex and gender differences. Most thought that this was the first time that these types of differences in infectious disease susceptibility were being reported, but I educated them and the public about the 1918 H1N1 pandemic, 1968 H3N2 pandemic, 2009 H1N1 pandemic, H5N1 outbreaks, SARS‐CoV outbreak, MERS outbreak, and ongoing HIV epidemic, all of which showed profound sex differences.^46^, ^47^, ^48^ I provided examples in which men suffered worse outcomes than women (e.g., 1918 H1N1, SARS, MERS, and SARS‐CoV‐2), and other examples in which women suffered worse outcomes than men for reasons including but not limited to pregnancy increasing the severity of viral outcomes (e.g., 1968 H3N2, 2009 H1N1, H5N1, and HIV). The media, the public, and my colleagues listened, and I received the blue check on Twitter, illustrating that I was a trusted expert.

As my laboratory shut down research during the worst of the pandemic, we began adapting our existing protocols for influenza viruses to SARS‐CoV‐2. We began testing immune responses to SARS‐CoV‐2 in patients who recovered from infection and showed that despite men and older aged individuals being more likely than women or younger aged individuals to be hospitalized with severe disease, they were mounting greater antibody responses to SARS‐CoV‐2^49^ and others were reporting the same observations.^50^ Greater antibody responses in males following SARS‐CoV‐2 infection might induce potent inflammatory responses, as observed for SARS‐CoV,^36^ and contribute to immune‐mediated pathology. I then began working in large teams of scientists to analyze data from electronic medical records^51^ and develop animal models to interrogate whether replicating virus, inflammatory immune responses, or both contributed to worse outcomes in males.^52^ We knew from our studies of H1N1 influenza viruses that disease can be caused by either an inability to resist a virus (i.e., control virus replication) or an inability to tolerate the damage caused by immune responses to a virus (i.e., immune‐mediated pathology).^25^, ^26^ The hypothesis that severe COVID‐19 outcomes were immune‐mediated, rather than mediated by replicating virus, was gaining empirical support and both our animal models and clinical data suggested that immune‐mediated inflammation was the cause of worse SARS‐CoV‐2 outcomes in males than females. At this time, we also began recognizing that SARS‐CoV‐2 was more likely to cause hospitalization in pregnant than non‐pregnant women and we collected samples, evaluated immune responses, and showed that infection caused heightened systemic inflammatory responses, including IL‐1β, and impaired antibody responses in pregnant women infected with SARS‐CoV‐2.^53^

For decades, I had struggled to get funding for sex differences and pregnancy research. Several years ago, it was the National Institutes of Health (NIH) Office of Research on Women's Health and their Specialized Centers of Research Excellence in sex differences program that gave me the break that I needed to study sex and age differences in vaccine‐induced immunity against influenza in human populations and animal models. During the early days of the pandemic, my collaborators and I leveraged our Center to get emergency funds to translate our knowledge about sex and age differences in responses to influenza vaccines,^54^ to studies of SARS‐CoV‐2 vaccines.^55^ Through support from the leadership at Johns Hopkins, I was asked to lead an application for an NIH‐funded COVID‐19 Serology Center of Excellence with another female clinician–scientist at Johns Hopkins University. Together, as multi‐principal investigators, we got a COVID‐19 Serology Center of Excellence funded; one of eight in the United States, and the only Center led solely by women. We leveraged this Center to support the efforts of junior scientists and clinicians, and we began applying for funding to support complementary efforts (e.g., to study how pregnancy affects vaccine‐induced immunity and protection). My laboratory members showed a work ethic and commitment that made me proud. We began publishing at a rate unrealized prior to the pandemic. Junior faculty, postdocs, and students from my group were finding themselves selected for talks at major conferences. The public and scientific community, alike, wanted to understand disparities between the sexes.

At home, my teenage daughters persevered, and I got to know them in new and different ways. I had never stayed home with them, and now I was home more than ever. I binge‐watched shows, exercised, laughed, and studied with them. My husband, whose career trajectory was steeper than mine, was there to take on the family responsibilities in major ways to give me the freedom to work and manage my growing laboratory and budgets. Once we were all vaccinated, I began visiting with my parents more and was saddened by how hard the pandemic was for older adults as routine checkups, surgeries, and functioning outside of the home became increasingly more difficult. In fact, my parents both got SARS‐CoV‐2 after receiving their vaccines—my mom remained asymptomatic, whereas my dad was admitted into the ICU, ventilated, and survived COVID‐19, only to come back stronger and more immune.

We each have a story to tell from our experiences during the pandemic. My story is one of perseverance, skill, and luck. It took a pandemic for the world to notice sex differences in the pathogenesis of viruses and responses to vaccines. As Pasture lamented, “chance favors the prepared mind.” With over two decades of preparation, when the time came, I was ready.

## CONFLICT OF INTEREST

None.

## Data Availability

Data sharing not applicable to this article as no datasets were generated or analysed during the current study
